# The mediating effect of DNA methylation in the association between maternal sleep during pregnancy and offspring adiposity status: a prospective cohort study

**DOI:** 10.1186/s13148-022-01284-w

**Published:** 2022-05-20

**Authors:** Min Meng, Yanrui Jiang, Jianfei Lin, Jun Zhang, Guanghai Wang, Qi Zhu, Qingmin Lin, Fan Jiang

**Affiliations:** 1 Department of Developmental and Behavioral Pediatrics, Pediatric Translational Medicine Institute, Shanghai Children’s Medical Center, School of Medicine, Shanghai Jiao Tong University, 1678 Dong Fang Road, Shanghai, 200127 China; 2 Ministry of Education-Shanghai Key Laboratory of Children’s Environmental Health, Xinhua Hospital, School of Medicine, Shanghai Jiao Tong University, Shanghai, 200092 China; 3 School of Public Health, Shanghai Jiao Tong University, Shanghai, 200025 China; 4 Shanghai Center for Brain Science and Brain-Inspired Technology, Shanghai, 201602 China; 5 School of Life Science and Biotechnology, Shanghai Jiao Tong University, 800 Dong Chuan Road, Shanghai, 200240 China

**Keywords:** Sleep midpoint, Late pregnancy, DNA methylation, Cord blood, Childhood adiposity

## Abstract

**Background:**

Childhood overweight/obesity is a global public health concern. It is important to identify its early-life risk factors. Maternal poor sleep is common in late pregnancy, and previous studies indicated that poor sleep may influence the offspring’s adiposity status. However, very few studies in humans investigated the effect of the different sleep parameters (sleep quantity, quality, and timing) on the offspring’s adiposity indicators, and long-term studies are even more scarce. In addition, the underlying mechanism remains unclear. The present study therefore aimed to examine the association between the three maternal sleep dimensions in the late pregnancy and the offspring adiposity indicators and to explore the potential mediating effect of the cord blood DNA methylation in the above association.

**Methods:**

Included participants in the current study were 2211 healthy pregnant women with singleton gestation from the Shanghai Birth Cohort (SBC) and Shanghai Sleep Birth Cohort (SSBC). Maternal nighttime sleep duration, quality, and midpoint (an indicator of circadian rhythm) were assessed by the same instrument in both cohorts during late pregnancy, and the offspring’s body mass index (BMI) and subcutaneous fat (SF) were measured at 2 years old. Additionally, in 231 SSBC samples, the genome-wide DNA methylation levels were measured using the Illumina Infinium Methylation EPIC BeadChip. The multivariate linear regression was used to determine the associations between the maternal sleep parameters and the offspring adiposity indicators. The epigenome-wide association study was conducted to identify the maternal sleep-related CpG sites. The mediation analysis was performed to evaluate the potential intermediate role of DNA methylation in the association between maternal sleep and offspring adiposity indicators.

**Results:**

The mean maternal nighttime sleep duration and the sleep midpoint for combined cohorts were 9.24 ± 1.13 h and 3.02 ± 0.82, respectively, and 24.5% of pregnant women experienced poor sleep quality in late pregnancy. After adjusting for the covariates, the maternal later sleep midpoint was associated with the increased SF in offspring (Coef. = 0.62, 95% CI 0.37–0.87, *p* < 0.001) at 2 years old. However, no significant associations of the nighttime sleep duration or sleep quality with the offspring adiposity indicators were found. In the SSBC sample, 45 differential methylated probes (DMPs) were associated with the maternal sleep midpoint, and then, we observed 10 and 3 DMPs that were also associated with the offspring’s SF and BMI at 2 years, of which cg04351668 (*MARCH9*) and cg12232388 significantly mediated the relationship of sleep midpoint and SF and cg12232388 and cg12225226 mediated the sleep midpoint–BMI association, respectively.

**Conclusions:**

Maternal later sleep timing in late pregnancy was associated with higher childhood adiposity in the offspring. Cord blood DNA methylation may play a mediation role in that relationship.

**Supplementary Information:**

The online version contains supplementary material available at 10.1186/s13148-022-01284-w.

## Introduction

For nearly half a century, the rapidly increasing prevalence of childhood overweight/obesity has raised worldwide concern [1]. Mounting evidence has demonstrated that childhood obesity, often tracking into adulthood [2], can increase risks of numerous adverse consequences in later life, such as metabolic syndrome, cardiovascular diseases, type 2 diabetes, and some cancers [3, 4]. Thus, it is important to identify early-life risk factors for childhood obesity.

Based on the developmental plasticity hypothesis [5, 6], certain prenatal factors, such as maternal nutrition [7], physical activity [8], and metabolic diseases [9], may have long-term effects on offspring’s obesity. In consideration of the well-established sleep-obesity link in children and adults [10, 11], and the high prevalence of poor sleep during pregnancy, especially in late pregnancy [12], researchers began to investigate the effect of maternal sleep on the offspring’s weight status and development. It has been reported that poor maternal sleep during the late pregnancy was associated with adverse outcomes in the fetus, such as preterm birth and low birth weight [13]. However, few studies have examined the long-term effect of poor maternal sleep in late pregnancy on the offspring’s adiposity status.

Sleep is characterized by multiple dimensions, such as sleep quantity, quality, and timing (an indicator of the circadian rhythm) [14]. One recent study with two cohorts investigated the relationship between maternal sleep quantity and the offspring’s adiposity and found that the shorter total sleep duration during the mid-term pregnancy significantly increased the offspring's adiposity; however, this association was not observed in the Rhea cohort (during late pregnancy) [15]. Besides insufficient sleep duration, poor sleep quality is also most complained during pregnancy [12]. Animal studies found higher body weight, higher lipid level, decreased glucose tolerance, and increased insulin resistance in male offspring when maternal mice were exposed to the gestational sleep fragmentation (one index of sleep quality) [16, 17], but no human evidence was available. Similarly, some emerging animal experiments indicated that the gestational chronodisruption or sleep circadian disruption induced deleterious effects in male rat offspring’s glucose homeostasis and adipose tissue function [18, 19], whereas few studies have been conducted in humans. In addition, lacking multidimensional assessments of sleep in previous studies made it unclear whether the associations vary between different sleep parameters and the offspring adiposity status.

Increasing evidence indicates that epigenetic modification, which regulates gene expression without the changes in the DNA sequence, plays a pivotal role in developmental plasticity [20]. The epigenetic mechanism mainly includes DNA methylation, histone modification, and regulation by non-coding RNAs, among which the DNA methylation is the best-studied and most common mechanism by the intense activity of the DNA methyltransferases during the early developmental period [21], and is also more stable and may have long-term effect on the offspring [22]. Animal model showed that the sleep fragmentation [17, 23] during the late gestation altered a large proportion of the DNA methylations in obesity and metabolic pathways, which were associated with obesity and metabolic syndrome in the offspring of 24 weeks old. However, no human studies have been conducted to examine the altered DNA methylation in the offspring associated with maternal sleep (e.g., quantity, quality, and timing) during late pregnancy.

To fill these research gaps, we aimed to examine the association between the three dimensions of maternal sleep (nighttime sleep duration, quality of sleep, and sleep midpoint between bedtime and get-up time) in the late pregnancy and the offspring adiposity status in a large prospective birth cohort and then used the epigenome-wide association study (EWAS) to explore whether DNA methylation may serve as a potential mechanism in the association between maternal sleep and offspring adiposity indicators.

## Methods

This study is reported according to Strengthening the Reporting of Genetic Association studies guideline, an extension of Strengthening the Reporting of Observational studies in Epidemiology statement [24]. The checklist could be found in Additional file 1: Table S1.

### Participants

The Shanghai Key Laboratory of Children’s Environmental Health conducted two ongoing birth cohorts: Shanghai Birth Cohort (SBC) and Shanghai Sleep Birth Cohort (SSBC) [25, 26]. The SBC aimed to investigate the effect of early-life exposure to environmental and behavioral factors on the child’s health, whereas the SSBC was more focused on the effect of sleep disturbances on the children’s growth and development. Details have been described previously. Briefly, the couples who aged 20–45 years, with at least one of the couple having a registered Shanghai resident, planned to obtain prenatal care and give birth at the participating hospitals, and stayed in Shanghai at least for 2 years were recruited into the cohorts from 2012 to 2016. Only singleton gestation women without any pregnancy complications were included in the present study. All measurements of the two cohorts used in the current study were designed according to the same protocol [25, 26]. To further explore the potential epigenetic mechanism, the cord blood DNA methylation analysis was performed from the SSBC, whose recruitment was conducted in the Renji Hospital from May 2012 to July 2013 (Additional file 2: Figure S1). Ethical approvals for the study were obtained from the ethics committee of the Shanghai Xinhua Hospital (XHEC-C-2013-001) and Shanghai Children's Medical Center (SCMCIRB-2012033). Written informed consent was obtained from each participant of this study.

### Main variables

#### Maternal sleep parameters

Maternal sleep parameters were measured during the late pregnancy via the following questions: (1) “During the past month, what time do you usually go to bed at night and get-up in the morning?” and the nighttime sleep duration was calculated as the interval from bedtime to get-up time; (2) “During the past month, how would you rate your sleep quality overall?” and a four-point Likert-type score from 0 to 3 (0 = very good, 1 = fairly good, 2 = fairly bad, and 3 = very bad) was used, and then, we defined 0 and 1 as good sleep quality and 2 and 3 as poor sleep quality. In addition, the sleep midpoint, a halfway point between bedtime and get-up time, was calculated to represent the sleep circadian rhythm [27]. We defined extreme values as greater than ± 6SD of the mean nighttime sleep duration and coded them as missing [28].

#### Offspring adiposity indicators

At the age of 2 years, children’s weight and recumbent length were measured using calibrated scales (Seca 335). The weight and length were measured to the nearest 0.01 kg and 0.1 cm, respectively. The body mass index (BMI) was calculated as the weight (kg) divided by the squared length (m^2^). Additionally, the triceps and subscapular skinfold thicknesses were measured on the left side of the body using the Harpenden skinfold caliper and then were added together to calculate the children’s subcutaneous fat (SF) at 2 years old. All of the indicators were measured twice by the trained research staff according to the standard protocol of WHO [29], and the average values were used in further analysis.

#### DNA methylation

In the SSBC, after the delivery, 3–5 ml cord blood samples were immediately collected into the tubes containing ethylenediaminetetraacetic acid preservative, stored at 4 °C until transported to the laboratory. Subsequently, the samples were stored in − 80 °C until further processing for this study. The genome-wide DNA methylation was measured using the Illumina Infinium Human Methylation EPIC BeadChip array (Illumina, San Diego, CA, USA) that covers over 850,000 CpG loci across the whole human genome. The genome DNA was isolated from the whole blood sample and converted to bisulfite on 96-well plates. Following a standard protocol provided by Illumina, the bisulfite-converted DNA was hybridized to the array BeadChip, and the arrays were then scanned by the Illumina iScan scanner.

The methylation data were processed using the Chip Analysis Methylation Pipeline (ChAMP) package [30]. Firstly, the ChAMP loaded the raw image data from the IDAT files. The probes and samples were filtered based on the following criteria [31]: (1) probes with a detection *p*-value of > 0.01 in at least one sample; (2) probes with a bead count of < 3 in at least 5% of samples per probe; (3) non-CpG probes; (4) SNP-related probes; (5) multi-hit probes; and (6) probes on the X and Y chromosome. Then, using the beta mixture quantile dilation (BMIQ) method [32], the *β* values were calculated and normalized [*β* = methylated signal/(methylated signal + unmethylated signal)] to represent the methylation level of each CpG locus in each sample. The *β* values ranged from 0 (no methylation) to 1 (100% methylation).

### Covariates

According to a recently suggested principle of confounder selection [33], we selected several covariates that may influence the association between maternal sleep and childhood obesity, such as maternal age [34], education level [35], pre-pregnancy BMI [36], gestational weight gain [36], and the offspring’s sex. All the pregnant women enrolled in the present study were asked to complete a sociodemographic questionnaire containing delivery age, pre-pregnancy weight and height, and maternal educational level. And the weight during the last prenatal care and neonates’ sex were extracted from the hospital clinical records. The maternal pre-pregnancy BMI was calculated from the self-reported weight and height and then was categorized into underweight (< 18.5 kg/m^2^), normal (18.5–24.9 kg/m^2^), and overweight (≥ 25.0 kg/m^2^) using the recommendations of World Health Organization. The gestational weight gain (GWG) was calculated as the pre-pregnancy weight subtracted from the last clinically recorded weight and then was classified into inadequate, adequate, and excessive GWG according to the 2009 Institute of Medicine (IoM) guidelines [37].

### Statistical analysis

Basic characteristics of mother and offspring were described with mean ± SD or frequency (percentage). The difference of these characteristics between the included and excluded participants was tested using the two-sample *t*-test and Chi-square test for continuous variables and categorical variables, respectively.

**Fig. 1:**
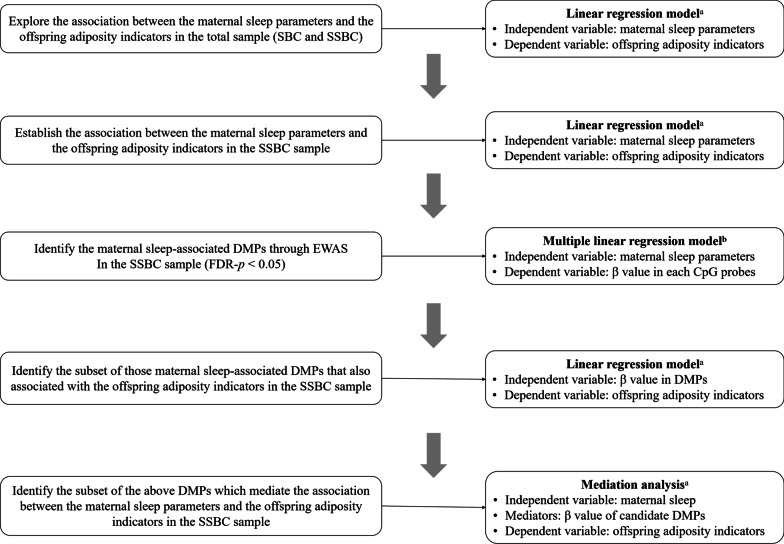
Statistical analysis workflow. DMPs, differential methylated probes; EWAS, epigenome-wide association study; FDR, false discovery rate; SBC, Shanghai birth cohort; SSBC, Shanghai sleep birth cohort. ^a^Adjusted for maternal age, education level, pre-pregnancy BMI, gestational weight gain, and offspring’s sex. ^b^Adjusted for maternal age, education level, pre-pregnancy BMI, gestational weight gain, offspring’s sex, batch effect, and cell type

Statistical analysis workflow is summarized in Fig. 1. Firstly, to determine the association between the three dimensions of the maternal sleep (nighttime sleep duration, sleep quality, and sleep midpoint) during the late pregnancy and the childhood adiposity status (BMI and SF) in the offspring, a linear regression model was used with adjusting for the first confounder set including the maternal age, education level, pre-pregnancy BMI, GWG, and the offspring sex both in the total sample and in the SSBC sample with available methylation data, respectively. Secondly, to identify the maternal sleep-related CpG sites, the EWAS analysis was performed using the multiple linear regression of *β*-value at each CpG site (dependent variable) on each maternal sleep parameter (independent variable), adjusting for the second confounder set including maternal age, education level, pre-pregnancy BMI, GWG, offspring sex, as well as batch effect by the ComBat function [38] and cell-type (neutrophils, monocytes, CD8 + T cells, CD4 + T cells, natural killer cells, and B cells) heterogeneity via the RefbaseEWAS [39]. To control the false discovery rate (FDR), the Benjamini–Hochberg correction method was applied for multiple testing, and the differential methylated probes (DMPs) were selected based on the FDR-corrected *p* values (FDR-*p*) of < 0.05. Thirdly, to further identify the subset of those maternal sleep-related DMPs that were also associated with the offspring’s adiposity indicators, a linear regression model with adjusting for the first confounder set was applied using the DMPs as independent variables and offspring’s adiposity indicators as dependent variables. Finally, according to the new procedures and recommendations of mediation analysis that a non-significant total effect does not necessarily indicate lack of mediation [40], a mediation analysis with adjusting for the first confounder set was performed to identify the mediators of the above DMPs in the association between the maternal sleep and the offspring’s adiposity indicators by the PROCESS procedure using 5000 bootstrap samples [41].

In consideration of the sex-specific effect of maternal sleep on the offspring weight outcomes in animal studies, the interactive effect of sex and the maternal sleep parameters on the offspring’s adiposity indicators was also performed. Firstly, we added an interaction term (maternal sleep * sex) in the multivariable linear regression models to assess the statistical interactive effect. Then, we conducted the sex-specific linear regression model to evaluate the biological interaction effect between maternal sleep and offspring sex [42]. Furthermore, the sensitivity analysis for the associations between the maternal sleep parameters and the offspring’s adiposity indicators was conducted by further extracting the subjects whose nighttime sleep duration was within − 4SD ~  + 4SD from the whole sample [28]. We further performed a multiple imputation using chained equations (MICE) with 100 imputed datasets to estimate missing values [43] and compared the results before and after the data imputation.

All analyses were conducted using the Stata/SE version 16.0, R 3.5.2, and the SPSS version 26.0.0, all statistical tests were two-sided, and the *p*-values (or FDR-*p*) of < 0.05 were considered as statistical significance (Fig. 1).

## Results

### Participants’ characteristics

After data cleaning, 2211 mother–child pairs from the SBC and SSBC with all the three maternal sleep parameters and the two offspring’s adiposity indicators were analyzed. From the SSBC, 231 participants with cord blood DNA methylation data with 731,843 probes were included in the EWAS analysis (Additional file 2: Figure S1). Compared with participants who were included in the present study, the participants who were excluded were more likely to have an older delivery age, overweight/obesity status before pregnancy, and inadequate GWG during pregnancy (Additional file 2: Table S1).

In the total sample (Table 1), the average delivery age of the mothers was 28.54 ± 3.54 years, 91.8% of mothers obtained a college or above education level, 10.2% mothers had pre-pregnancy overweight status, and 42.6% mothers experienced excessive GWG. During the late pregnancy, the maternal nighttime sleep duration was 9.24 ± 1.13 h, the sleep midpoint was 3.02 ± 0.82, and 24.5% of pregnant women suffered from poor sleep quality. The offspring’s BMI and SF at 2 years old were 16.52 ± 1.39 kg/m^2^ and 21.27 ± 4.17 mm, respectively.

**Table 1:** Characteristics of mother and offspring in the cohorts

	Cohorts of Shanghai key laboratory		
	Overall (*N* = 2211)	SBC (*N* = 1957)	SSBC (*N* = 254)
	Mean ± SD/*n* (%)	Mean ± SD/*n* (%)	Mean ± SD/*n* (%)
*Mother*			
Age at delivery (years)	28.54 ± 3.54	28.42 ± 3.57	29.47 ± 3.16
Education level			
High school or below	182 (8.2)	159 (8.1)	23 (9.1)
College or above	2029 (91.8)	1798 (91.9)	231 (90.9)
Pre-pregnancy BMI (kg/m^2^)			
< 18.5	360 (16.3)	312 (15.9)	48 (18.9)
18.5–25	1625 (73.5)	1465 (74.9)	160 (63.0)
≥ 25	226 (10.2)	180 (9.2)	46 (18.1)
Gestational weight gain			
Inadequate	394 (17.8)	368 (18.8)	26 (10.2)
Adequate	875 (39.6)	763 (39.0)	112 (44.1)
Excessive	942 (42.6)	826 (42.2)	116 (45.7)
Nighttime sleep duration (h)	9.24 ± 1.13	9.22 ± 1.12	9.44 ± 1.16
Sleep quality			
Good	1668 (75.5)	1494 (76.4)	174 (68.8)
Poor	541 (24.5)	462 (23.6)	79 (31.2)
Sleep midpoint	3.02 ± 0.82	3.00 ± 0.82	3.21 ± 0.80
*Offspring*			
Sex			
Boy	1137 (51.4)	1007 (51.5)	130 (51.2)
Girl	1074 (48.6)	950 (48.5)	124 (48.8)
BMI at 24 months (kg/m^2^)	16.52 ± 1.39	16.57 ± 1.39	16.03 ± 1.32
SF at 24 months (mm)	21.27 ± 4.17	20.40 ± 3.06	27.27 ± 5.63

*BMI* body mass index; *SF* subcutaneous fat; *SBC* Shanghai birth cohort; *SSBC* Shanghai sleep birth cohort

### Associations of maternal sleep parameters and offspring’s adiposity indicators

After adjusting for the covariates, we observed that later maternal sleep midpoint during late pregnancy was significantly associated with the increased offspring’s SF at 2 years old (Coeff. = 0.62, 95% CI 0.37–0.87, *p* < 0.001). However, nighttime sleep duration and sleep quality were not significantly associated with the offspring adiposity indicators (Table 2). Similar results were also found in the SSBC sample with available methylation data (Table 3).

**Table 2:** The associations between maternal sleep parameters during late pregnancy and offspring adiposity indicators at 2 years old in the total sample

	BMI (*N* = 2167)		SF (*N* = 1493)	
	Coef. (95% CI)	*p*	Coef. (95% CI)	*p*
*Model 1*				
Nighttime sleep duration	**0.05 (0.00, 0.11)**	**0.039**	− 0.09 (− 0.27, 0.10)	0.355
Sleep quality				
Good	Ref		Ref	
Poor	− 0.10 (− 0.24, 0.04)	0.154	0.09 (− 0.42, 0.60)	0.739
Sleep midpoint	− 0.02 (− 0.09, 0.06)	0.667	**0.60 (0.35, 0.86)**	** < 0.001**
*Model 2*				
Nighttime sleep duration	0.05 (− 0.00, 0.10)	0.076	− 0.01 (− 0.20, 0.17)	0.880
Sleep quality				
Good	Ref		Ref	
Poor	− 0.05 (− 0.19, 0.08)	0.449	0.08 (− 0.42, 0.58)	0.746
Sleep midpoint	− 0.03 (− 0.10, 0.04)	0.467	**0.62 (0.37, 0.87)**	** < 0.001**

*BMI* body mass index; *SF* subcutaneous fat

Ref.: women with good sleep quality were selected as reference group

Model 1: unadjusted

Model 2: adjusted for maternal age, education level, pre-pregnancy BMI, gestational weight gain, and offspring’s sex

The bold values indicate statistical significance of *p* < 0.05

**Table 3:** The association between the maternal sleep parameters during late pregnancy and the offspring adiposity indicators at 2 years old in the SSBC sample

	BMI (*N* = 213)		SF (*N* = 189)	
	Coef. (95% CI)	*p*	Coef. (95% CI)	*p*
*Model 1*				
Nighttime sleep duration	0.06 (− 0.09, 0.21)	0.442	0.59 (− 0.07, 1.24)	0.080
Sleep quality				
Good	Ref		Ref	
Poor	− 0.23 (− 0.61, 0.15)	0.226	0.02 (− 1.69, 1.74)	0.978
Sleep midpoint	0.04 (− 0.19, 0.27)	0.732	**1.38 (0.41, 2.35)**	**0.006**
*Model 2*				
Nighttime sleep duration	0.04 (− 0.11, 0.19)	0.623	0.42 (− 0.23, 1.08)	0.207
Sleep quality				
Good	Ref		Ref	
Poor	− 0.21 (− 0.59, 0.17)	0.283	0.14 (− 1.59, 1.88)	0.871
Sleep midpoint	− 0.02 (− 0.25, 0.21)	0.864	**1.04 (0.06, 2.03)**	**0.038**

*BMI* body mass index; *SF* subcutaneous fat; *SSBC* Shanghai sleep birth cohort

Ref.: women with good sleep quality were selected as reference group

Model 1: unadjusted

Model 2: adjusted for maternal age, education level, pre-pregnancy BMI, gestational weight gain, and offspring sex

The bold values indicate statistical significance of *p* < 0.05

### Maternal sleep-specific associations with the cord blood CpG sites

**Fig. 2:**
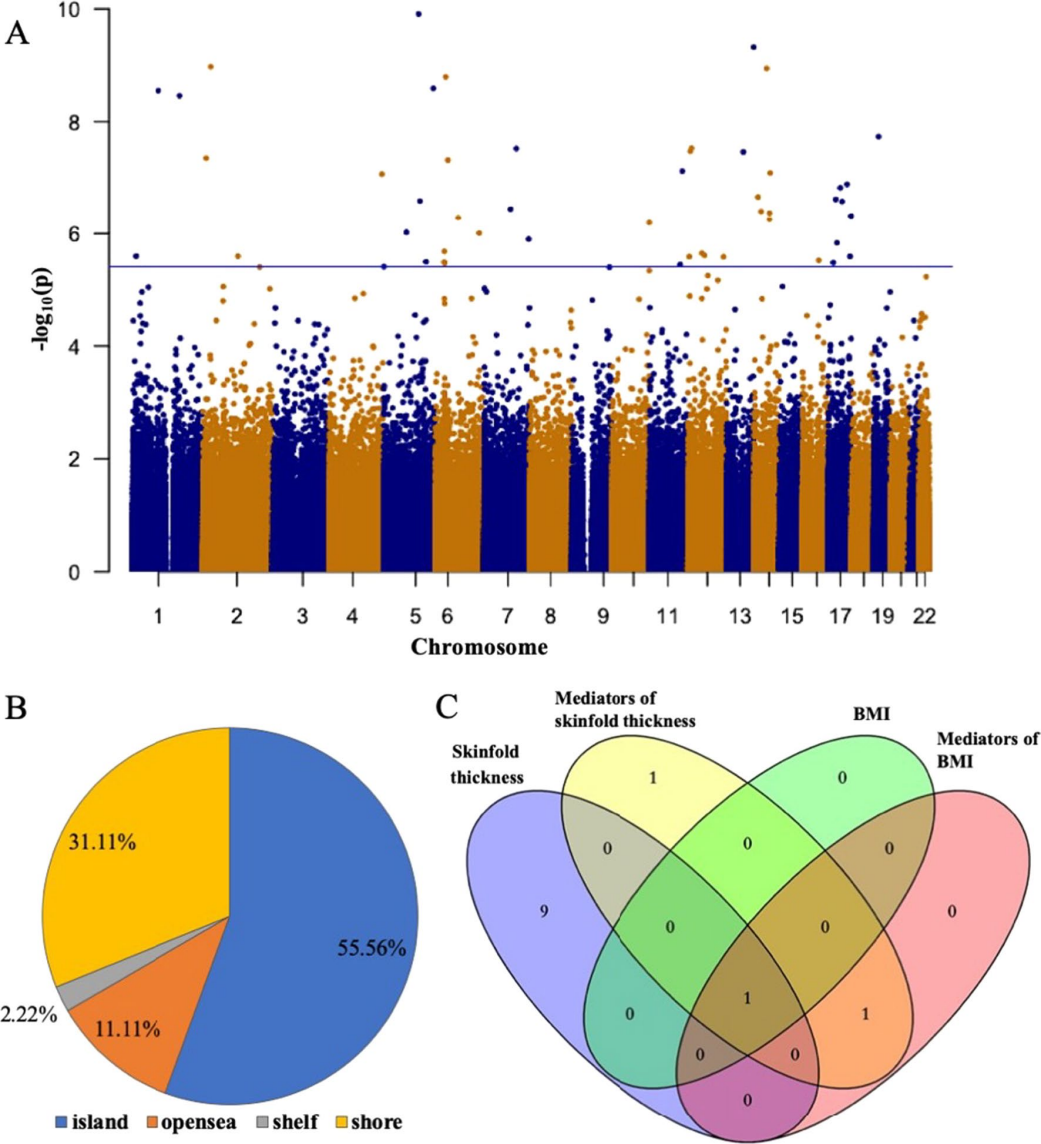
Cord blood DMPs associated with maternal sleep midpoint during late pregnancy. **A** Manhattan plot showing the distribution of DMPs associated with maternal sleep midpoint in chromosome. The blue line represents the threshold for multiple testing (FDR-*p* < 0.05). DMPs, differential methylated probes. **B** Pie chart showing the distribution of maternal sleep midpoint-related DMPs across CpG island region. **C** Venn diagram showing the overlap of maternal sleep midpoint-related DMPs associated with offspring body mass index (BMI) and subcutaneous fat (SF)

From the EWAS analysis, after adjusting for confounders, 45 DMPs that were related to maternal sleep midpoint mapping to 38 genes remained statistically significant (FDR-*p* < 0.05) (Additional file 2: Table S3). The distribution of these DMPs in chromosome is shown in Fig. 2A. In total, 97.8% of the selected DMPs showed hypermethylation, and 55.6% of them were located in the island region (Fig. 2B). However, we did not find any DMPs significantly associated with the maternal nighttime sleep duration or sleep quality during the late pregnancy.

### Associations of the maternal sleep-associated DMPs with the offspring adiposity indicators

We found that 10 maternal sleep midpoint-associated DMPs, mapping to *MARCH9*, *SLC29A1*, *RHBDL3*, *NLK*, *C14orf147*, *KPNA2*, *ABHD8*, *DDX20*, and *EME1* genes, were also positively associated with the offspring’s SF. And three DMPs with one mapping to *SFRS3* gene were negatively associated with the offspring’s BMI (Table 4). Moreover, there was an overlap that the methylation level of cg12232388 was associated with both higher SF and BMI at 2 years old (Table 4 and Fig. 2C).

**Table 4:** The association between DMPs related to maternal sleep midpoint during late pregnancy and offspring adiposity indicators at 2 years old in the SSBC sample^a^

	BMI (*N* = 191)		SF (*N* = 169)	
	Coef. (95% CI)	*p*	Coef. (95% CI)	*p*
*Maternal sleep midpoint-related DMPs (gene)*				
cg04351668 (*MARCH9*)	2.96 (− 1.40, 7.32)	0.182	**33.21 (13.92, 52.51)**	**0.001**
cg04783204 (*SLC29A1*)	4.81 (− 9.11, 18.73)	0.496	**101.68 (39.71, 163.65)**	**0.001**
cg04794832 (*RHBDL3*)	8.76 (− 8.13, 25.65)	0.307	**99.36 (28.98, 169.74)**	**0.006**
cg08743881 (*NLK*)	2.63 (− 6.36, 11.61)	0.565	**48.24 (5.91, 90.57)**	**0.026**
cg09951570 (*C14orf147*)	1.54 (− 12.85, 15.92)	0.833	**69.99 (3.18, 136.79)**	**0.040**
cg14898140 (*KPNA2*)	4.85 (− 6.51, 16.21)	0.401	**66.54 (15.14, 117.94)**	**0.011**
cg14935078 (*ABHD8*)	3.46 (− 2.63, 9.54)	0.264	**31.70 (4.17, 59.22)**	**0.024**
cg17427781 (*DDX20*)	3.69 (− 5.56, 12.95)	0.432	**58.40 (18.53, 98.26)**	**0.004**
cg18310007 (*EME1*)	4.72 (− 13.69, 23.13)	0.614	**102.58 (20.23, 184.93)**	**0.015**
cg12232388	**8.72 (1.52, 15.92)**	**0.018**	**74.80 (43.36, 106.24)**	** < 0.001**
cg12225226	** − 7.90 (− 13.66, − 2.14)**	**0.007**	3.00 (− 22.05, 28.04)	0.813
cg18489012 (*SFRS3*)	** − 17.41 (− 32.12, − 2.70)**	**0.021**	− 12.82 (− 80.33, 54.69)	0.708

*BMI* body mass index; *DMPs* differential methylated probes; *SF* subcutaneous fat; *SSBC* Shanghai sleep birth cohort

^a^Adjusted for maternal age, education level, pre-pregnancy BMI, gestational weight gain, and offspring’s sex

The bold values indicate statistical significance of *p* < 0.05

### Mediating effect of the maternal sleep-associated DMPs

In a further mediation analysis, the maternal sleep midpoint-associated DMPs that were also associated with the offspring’s adiposity indicators were considered as potential mediators. In the unadjusted model, the cg04351668 (*MARCH9*) and cg12232388 showed significant mediating effects in the association between maternal sleep midpoint during the late pregnancy and the offspring’s SF at 2 years old. After adjusting for the covariates, the positive mediating effects remained significant (Table 5).

**Table 5:** The mediating effect of the selected DMPs^a^ between maternal sleep midpoint during late pregnancy and offspring adiposity indicators at 2 years old in the SSBC sample

	BMI (*N* = 191)				SF (*N* = 169)			
	Effect (a*b)	SE	LLCI	ULCI	Effect (a*b)	SE	LLCI	ULCI
*Model 1*								
cg04351668 (*MARCH9*)	0.056	0.053	− 0.012	0.187	**0.408**	**0.271**	**0.019**	**1.064**
cg18310007 (*EME1*)	0.021	0.055	− 0.071	0.151	0.446	0.298	− 0.002	1.114
cg12232388	**0.075**	**0.048**	**0.004**	**0.189**	**0.704**	**0.264**	**0.245**	**1.262**
cg12225226	** − 0.141**	**0.057**	** − 0.267**	** − 0.045**	− 0.140	0.181	− 0.578	0.140
*Model 2*								
cg04351668 (*MARCH9*)	0.048	0.050	− 0.017	0.178	**0.368**	**0.282**	**0.004**	**1.060**
cg18310007 (*EME1*)	0.021	0.050	− 0.067	0.134	0.328	0.251	− 0.071	0.899
cg12232388	**0.089**	**0.054**	**0.011**	**0.220**	**0.841**	**0.320**	**0.289**	**1.528**
cg12225226	** − 0.145**	**0.058**	** − 0.277**	** − 0.052**	− 0.118	0.198	− 0.612	0.185

*BMI* body mass index; *DMPs* differential methylated probes; *LLCI* lower limit confidence interval; *SF* subcutaneous fat; *SE* standard error; *ULCI* upper limit confidence interval; *SSBC*

^a^DMPs were related with maternal sleep midpoint during late pregnancy, and were also associated with offspring’s adiposity indicators

a*b shows the mediating effect of DMPs in the maternal sleep midpoint–BMI and maternal sleep midpoint–SF relationships, of which “a” shows the coefficient of the association between maternal sleep midpoint and DMPs, and “b” shows the coefficient of the association between DMPs and offspring adiposity indicators

Model 1: unadjusted

Model 2: adjusted for maternal age, education level, pre-pregnancy BMI, gestational weight gain, and offspring’s sex

The bold values indicate statistical significance of *p* < 0.05

Although there was no significant effect of the maternal sleep midpoint during the late pregnancy on the offspring’s BMI at 2 years old, two DMPs (cg12232388, and cg12225226) showed significant mediating effects between them after adjusting for the covariates (Table 5).

### Sensitivity analysis

We did not find a significant interactive effect of the maternal sleep parameters with the offspring’s sex on the offspring’s adiposity indicators even though the effect size of maternal sleep midpoint on offspring’s SF was larger in female offspring (Coef. = 0.78 vs Coef. = 0.49) (Additional file 2: Table S3). After limiting participants whose nighttime sleep durations were within -4SD ~  + 4SD, the results did not change much (Additional file 2: Table S4). Furthermore, there were about 0.22% ~ 33.09% missing data for each variable, and we found the results were similar before and after data imputation (Additional file 2: Tables S5–S7).

## Discussion

To the best of our knowledge, this was the first large birth cohort study to explore the long-term effect of maternal sleep during the late pregnancy, including the sleep quantity, quality, and timing on the offspring adiposity status. We also used the EWAS to determine the potential epigenetic mechanism of the relationship in a relatively small sample. Our study provided new insights that the maternal later sleep timing during late pregnancy was significantly associated with the increased offspring’s SF at 2 years old. In addition, we identified 45 cord blood DMPs mapping to 38 genes related to the maternal sleep midpoint, and approximately 98% of them were hypermethylated. Moreover, 10 and 3 maternal sleep midpoint-related DMPs were also associated with the offspring’s SF (*MARCH9, SLC29A1*, *RHBDL3*, *NLK*, *C14orf147*, *KPNA2*, *ABHD8*, *DDX20*, and *EME1*) and BMI (*SFRS3*) at 2 years, of which cg04351668 (*MARCH9*) and cg12232388 had significant mediating effects in the relationship of sleep midpoint and SF and cg12232388 and cg12225226 significantly mediated the sleep midpoint–BMI association, respectively. However, we did not observe a significant relationship of the maternal nighttime sleep duration and quality with the offspring adiposity status, and we also did not find any significant cord blood DNA methylation sites.

Emerging studies showed that sleep timing or midpoint, as a maker of circadian rhythm, played an important role in the adiposity. Later sleep in adulthood was significantly associated with the higher BMI status, and “later sleepers” whose sleep midpoint was ≥ 5:30 AM were more likely to have obesity conditions [27, 44]. Similarly, the adolescents with evening chronotypes (sleeping late) had significantly higher BMI level when compared to those with the definitely morning chronotype [45]. Moreover, a recent study in adults also indicated that the individuals with sleep end time of 1 h later had a 1.64% increase in their body fat percentage [46]. Our study was the first to show that the maternal later sleep midpoint significantly predicted higher SF in the offspring at 2 years old. We did not find any significant effect of the maternal sleep midpoint on the offspring’s BMI status, and the potential reason may be due to the overall weight gain lagging behind the fat accumulation which would be a much more sensitive outcome of the maternal sleep midpoint [47, 48]. The findings indicated that the negative impact of maternal later sleep on the offspring’s adiposity status may sustain to the early childhood. More studies should substantiate our findings in future.

We identified 45 cord blood DMPs that were associated with the maternal sleep midpoint during the late pregnancy, some of which were potentially associated with the offspring’s adiposity indicators. Specifically, a higher methylation level of the cg04351668 (*MARCH9*) and cg12232388 was associated with higher SF and also mediated the maternal sleep midpoint–SF relationship. In addition, although we did not find a significant overall association of maternal midpoint and offspring BMI, higher and lower methylation levels of the cg12232388 and cg12225226 were associated with higher BMI, respectively, and also mediated the maternal sleep midpoint–BMI relationship, which may be served as potential mediators. This outcomes indicated that the potentially relevant genes of the maternal sleep midpoint influencing on offspring’s adiposity outcomes may be due to the DNA methylation. However, the gene *MARCH9* on cg04351668 was only found to be involved in protein ubiquitination; meanwhile, we did not find genes on or nearest the other two DMPs; thus, more studies are needed to confirm our findings and to explore their roles in obesity. The cg08743881, mapped to *NLK*, was also positively associated with the offspring’s SF. The *NLK* gene, located in 17q11.2, contributed to various signaling pathways via modulating or interacting with the diverse transcription factors and also inhibited the expression of the adipogenic genes [49]. Higher methylation of the site in the offspring due to the maternal late sleep, therefore, would inhibit the *NLK* expression and then lead to fat accumulation in childhood.

Furthermore, we also identified some other maternal sleep midpoint-related DMPs that were associated with the genes regulating the pancreatic β-cell function and energy homeostasis, which may contribute to the occurrence and development of childhood obesity [50]. Specifically, the NPC2 (cg01680773) protein, a novel autocrine factor, is essential to prevent the switching of white adipocytes to a metabolically active state (similar to brown adipocytes), which is regarded as a therapeutic target of obesity, insulin resistance, and type 2 diabetes [51]; the *HMGN3* (cg19643841) expresses in all mice pancreatic endocrine cells and plays a key role in glucose homeostasis [52]; the *CDKN1B* (cg11728497), also known as p27(*KIP1*), regulates the cell motility and apoptosis, as well as the blood glucose level in the pancreatic beta cells; the *CADM1* (cg18603396) gene encodes membrane proteins that mediate synaptic assembly and regulates energy balance and weight status when it is expressed in the hypothalamus and hippocampus regions [53]; and the *EN2* (cg10481660) is identified as a novel obesity-related gene in some genome-wide association studies [54–56]. Previous studies observed the DNA methylation alterations of the core circadian genes that were resulted from the later sleep [57]. The wakefulness for a single night in shift workers increased the DNA methylation of *CRY1* and *PER1* in the adipose tissue [58, 59], and the later sleep midpoint in adolescents was also associated with the higher DNA methylation of *CRY2*, *PER1*, *RORB*, and *NR1D1* in the blood [60]. In the current study, the cg25173039 was mapped to the *CIPC* gene that inhibited the *CLOCK–BMAL1* activity and had a negative-feedback regulating effect of the circadian clock [57]. However, we did not find any significant relationship between the aforementioned maternal sleep midpoint-related DMPs and the 2 -year-old offspring’s SF or BMI, which may be due to the limited sample size in the present study. In future, more large cohort studies are needed to confirm the association between the DNA methylation sites mentioned above and offspring’s obesity status.

We did not find significant associations between the maternal nighttime sleep duration and sleep quality during the late pregnancy with the 2-year-old offspring’s adiposity indicators. We also did not identify any significant DMPs associated with the maternal nighttime sleep duration and sleep quality. However, the DNA methylation alterations resulting from insufficient sleep duration and poor sleep quality were reported in prior adult and adolescent researches. For example, even one-night acute sleep loss in adults resulted in 148 significant lipid metabolism-related differentially methylated regions in the subcutaneous adipose tissue [61]. Additionally, both objectively measured shorter sleep duration and higher sleep fragmentation in adolescents were associated with the DNA methylation of metabolism-related genes in the blood [62]. As specific to the intrauterine impact of maternal sleep, only animal studies were conducted and showed that the sleep fragmentation during the late gestation induced the offspring’s AdipoQ methylation modification along with the higher weight and other metabolic syndrome-like phenotypes [16, 63]. Animal research indeed plays an important role in providing insights into this research field [64]; however, many researchers realized that the conclusions drawn from animal studies cannot be simply transferred to human studies, due to the wide genetic heterogeneity and variety of metabolic pathways [65]. Therefore, more human studies are needed for substantiating the current findings.

## Limitation

Several potential limitations should be taken into consideration when interpreting the results of this study. Firstly, the majority of the mothers were well educated in our study, and therefore, the results might not be generalizable to the national population. Secondly, the sleep parameters were self-reported which may have brought the reporting bias. Future researches are needed to estimate the sleep parameters using objective methods, such as actiwatch. Thirdly, although we assessed BMI and SF indicators, direct measurements of fat mass and body fat distribution as well as blood biomarkers were not measured. Meanwhile, the potential measurement errors could also influence our findings. Fourthly, although the small sample for the EWAS may have been limited to finding more certain associations, our results showed that the later maternal sleep midpoint significantly predicted the increased offspring’s adiposity indicator, and additional larger cohort study was needed to confirm our findings. In addition, the DNA methylation was measured only in the cord blood, not fat-specific tissues, which may have attenuated the effect of maternal sleep during the late pregnancy on the DNA methylation in the offspring. Fifthly, in the current study, we only studied the offspring obesity-related outcomes at 2 years old and longer-term follow-up to evaluate whether the association persists or disappears was also needed. Moreover, we only included the normal pregnancy women, and it is also necessary to pay attention to those with sleep disorder in future. Finally, given the observational nature of our study, we could not exclude the influences by the residual and unmeasured or unknown confounding factors; additional larger and external cohort studies considering more relevant confounding factors were needed to confirm our findings.

## Conclusion

Our study provided the first human evidence that the cord blood DNA methylation mediated the relationship between the maternal sleep midpoint during the late pregnancy and the offspring’s adiposity status in childhood. Our findings highlighted that the DNA methylation alteration resulted from the later maternal sleep timing maybe a potential mechanism of the fetal programming of adiposity. Furthermore, larger cohort studies were needed to confirm our findings.

## Supplementary Information

**Additional file 1: Table S1.** The checklist from STROBE statement.

**Additional file 2: Table S1.** Comparison of the maternal and offspring’s characteristics between the included and excluded participants; **Table S2.** The maternal sleep midpoint-associated DMPs in cord blood in the SSBC sample; **Table S3**. The sex difference of the association between each maternal sleep parameter and each offspring’s adiposity indicator at 2 years old in total sample; **Table S4.** The sensitivity analysis of the association between each maternal sleep parameter and each offspring’s adiposity indicator at 2 years old in total sample. **Table S5.** The association between the maternal sleep parameters during late pregnancy and the offspring adiposity indicators at 2 years old in the total sample after multiple imputation; **Table S6.** The sex-specific association between each maternal sleep parameter and the offspring adiposity indicators at 2 years old in total sample after multiple imputation; **Table S7** The sensitivity analysis of the association between each maternal sleep parameter and the offspring adiposity indicators at 2 years old in total sample after imputation. **Figure S1.** Flowchart of the participants.

## Data Availability

The datasets used in the current study are available from the corresponding authors on reasonable request.

## Abbreviations

BMI: Body mass index
DMPs: Differential methylated probes
EWAS: Epigenome-wide association study
GWG: Gestational weight gain
SBC: Shanghai birth cohort
SF: Subcutaneous fat
SSBC: Shanghai sleep birth cohort
