# Supplementary material for: The mediating effect of DNA methylation in the association between maternal sleep during pregnancy and offspring adiposity status: a prospective cohort study
Source: Clin Epigenetics. 2022 May 20;14:66. doi: 10.1186/s13148-022-01284-w (PMC9123687; doi:10.1186/s13148-022-01284-w)
Supplement: Supplementary file 1 — Additional file 1: Table S1. The checklist from STROBE statement. [file 13148_2022_1284_MOESM1_ESM.docx]

# TABLE S1. the checklist from STROBE Statement

| **Item** | **Item number** | **STROBE Guideline** | **Page number** |
| --- | --- | --- | --- |
| **Title and Abstract** | 1 | (a) Indicate the study’s design with a commonly used term in the title or the abstract. | Page1 |
|  |  | (b) Provide in the abstract an informative and balanced summary of what was done and what was found. | Page2-3 |
| **Introduction** | | |  |
| *Background rationale* | 2 | Explain the scientific background and rationale for the investigation being reported. | Page4-5 |
| *Objectives* | 3 | State specific objectives, including any pre-specified hypotheses. | Page5 |
| **Methods** | | |  |
| *Study design* | 4 | Present key elements of study design early in the paper. | Page5 |
| *Setting* | 5 | Describe the setting, locations and relevant dates, including periods of recruitment, exposure, follow-up, and data collection. | Page6 |
| *Participants* | 6 | 1. **Cohort study –** Give the eligibility criteria, and the sources and methods of selection of participants. Describe methods of follow-up.   **Case-control study –** Give the eligibility criteria, and the sources and methods of case ascertainment and control selection. Give the rationale for the choice of cases and controls.  **Cross-sectional study –** Give the eligibility criteria, and the sources and methods of selection of participants. | Page6 |
|  |  | 1. **Cohort study –** For matched studies, give matching criteria and number of exposed and unexposed.   **Case-control study –** For matched studies, give matching criteria and the number of controls per case. |  |
| *Variables* | 7 | *(a)* Clearly define all outcomes, exposures, predictors, potential confounders, and effect modifiers. Give diagnostic criteria, if applicable. | Page6-7 |
| *Data sources measurement* | 8***** | *(a)* For each variable of interest, give sources of data and details of methods of assessment (measurement). Describe comparability of assessment methods if there is more than one group. | Page6-7 |
| *Bias* | 9 | *(a)* Describe any efforts to address potential sources of bias. | Page6 |
| *Study size* | 10 | Explain how the study size was arrived at. | Page9 |
| *Quantitative variables* | 11 | Explain how quantitative variables were handled in the analyses. If applicable, describe which groupings were chosen, and why. | Page8 |
| Statistical methods | 12 | (a) Describe all statistical methods, including those used to control for confounding. | Page8-9 and Figure1 |
|  |  | (b) Describe any methods used to examine subgroups and interactions. | Page8 and page9 |
|  |  | (c) Explain how missing data were addressed. | Page9 |
|  |  | 1. **Cohort study –** If applicable, explain how loss to follow-up was addressed.   **Case-control study –** If applicable, explain how matching of cases and controls was addressed.  **Cross-sectional study –** If applicable, describe analytical methods taking account of sampling strategy. | FigureS1 |
|  |  | (e) Describe any sensitivity analyses. | Page9 |
| **Results** | | |  |
| *Participants* | 13***** | 1. Report the numbers of individuals at each stage of the study – e.g., numbers potentially eligible, examined for eligibility, confirmed eligible, included in the study, completing follow-up, and analysed. | Page9 |
|  |  | (b) Give reasons for non-participation at each stage. | Figure S1 |
|  |  | (c) Consider use of a flow diagram. | Figure S1 |
| *Descriptive data* | 14***** | (a) Give characteristics of study participants (e.g., demographic, clinical, social) and information on exposures and potential confounders. | Page10 |
|  |  | (b) Indicate the number of participants with missing data for each variable of interest. | Page11 |
|  |  | 1. **Cohort study –** Summarize follow-up time, e.g. average and total amount. | FigureS1 |
| *Outcome data* | 15 ***** | **Cohort study-**Report numbers of outcome events or summary measures over time. | Figure S1 |
|  |  | **Case-control study –** Report numbers in each exposure category, or summary measures of exposure. |  |
|  |  | **Cross-sectional study –** Report numbers of outcome events or summary measures. |  |
| *Main results* | 16 | (a) Give unadjusted estimates and, if applicable, confounder-adjusted estimates and their precision (e.g., 95% confidence intervals). Make clear which confounders were adjusted for and why they were included. | Page10-11 |
|  |  | (b) Report category boundaries when continuous variables were categorized. | Page10 and Table1 |
|  |  | (c) If relevant, consider translating estimates of relative risk into absolute risk for a meaningful time period. |  |
| *Other analyses* | 17 | 1. Report other analyses done – e.g., analyses of subgroups and interactions, and sensitivity analyses. | Page11 |
| **Discussion** | | |  |
| *Key results* | 18 | Summarize key results with reference to study objectives. | Page11-12 |
| *Limitations* | 19 | Discuss limitations of the study, taking into account sources of potential bias or imprecision. Discuss both direction and magnitude of any potential bias. | Page 14-15 |
| *Interpretation* | 20 | Give a cautious overall interpretation of results considering objectives, limitations, multiplicity of analyses, results from similar studies, and other relevant evidence. | Page12-14 |
| *Generalizability* | 21 | Discuss the generalizability (external validity) of the study results. | Page14 |
| **Other Information** | | |  |
| *Funding* | 22 | Give the source of funding and the role of the funders for the present study and, if applicable, for the original study on which the present article is based. | Page15-16 |
