# Supplementary material for: The mediating effect of DNA methylation in the association between maternal sleep during pregnancy and offspring adiposity status: a prospective cohort study
Source: Clin Epigenetics. 2022 May 20;14:66. doi: 10.1186/s13148-022-01284-w (PMC9123687; doi:10.1186/s13148-022-01284-w)
Supplement: Supplementary file 2 — Additional file 2: Table S1. Comparison of the maternal and offspring’s characteristics between the included and excluded participants; Table S2. The maternal sleep midpoint-associated DMPs in cord blood in the SSBC sample; Table S3. The sex difference of the association between each maternal sleep parameter and each offspring’s adiposity indicator at 2 years old in total sample; Table S4. The sensitivity analysis of the association between each maternal sleep parameter and each offspring’s adiposity indicator at 2 years old in total sample. Table S5. The association between the maternal sleep parameters during late pregnancy and the offspring adiposity indicators at 2 years old in the total sample after multiple imputation; Table S6. The sex-specific association between each maternal sleep parameter and the offspring adiposity indicators at 2 years old in total sample after multiple imputation; Table S7 The sensitivity analysis of the association between each maternal sleep parameter and the offspring adiposity indicators at 2 years old in total sample after imputation. Figure S1. Flowchart of the participants. [file 13148_2022_1284_MOESM2_ESM.docx]

**Table S1 Comparation of the maternal and offspring characteristics between the included and excluded participants**

|  | Included (N = 2211) | Excluded (N = 1728) |  |
| --- | --- | --- | --- |
|  | Mean ± SD / n (%) | Mean ± SD / n (%) | *p* |
| **Maternal** |  |  |  |
| Age at delivery (years) | 28.54 ± 3.54 | 29.04 ± 3.73 | **<0.001** |
| Education level |  |  |  |
| High school or below | 182 (8.2) | 162 (9.3) | 0.232 |
| College or above | 2029 (91.8) | 1578 (90.7) |  |
| Pre-pregnancy BMI (kg/m^2^) |  |  |  |
| <18.5 | 360 (16.3) | 231 (13.7) | **0.027** |
| 18.5-25 | 1625 (73.5) | 1256 (74.3) |  |
| ≥25 | 226 (10.2) | 203 (12.0) |  |
| Gestational weight gain |  |  |  |
| Inadequate | 394 (17.8) | 422 (25.2) | **<0.001** |
| Adequate | 875 (39.6) | 551 (32.8) |  |
| Excessive | 942 (42.6) | 703 (42.0) |  |
| **Offspring** |  |  |  |
| Sex |  |  |  |
| Boy | 1137 (51.4) | 687 (50.5) | 0.597 |
| Girl | 1074 (48.6) | 673 (49.5) |  |

BMI, body mass index.

**Table S2 The maternal sleep midpoint associated DMPs in cord blood in the SSBC sample (N = 231)**^a^

|  | Coef. | SE | *p* | FDR-*p* | Chromosome | Gene | Feature | Gene function^*^ |
| --- | --- | --- | --- | --- | --- | --- | --- | --- |
| cg26527775 | 1.91E-02 | 2.43E-03 | 1.97E-13 | 1.44E-07 | 12 | *ZNF268* | 5'UTR | Nucleic acid binding,DNA binding,transcription factor activity, sequence-specific DNA binding,metal ion binding |
| cg15890707 | 1.96E-02 | 2.62E-03 | 1.74E-12 | 6.36E-07 | 5 | *DUSP1* | Body | MAPK signaling pathway |
| cg13036855 | 1.51E-02 | 2.17E-03 | 3.70E-11 | 9.02E-06 | 3 | *CHDH* | TSS1500 | Glycine, serine and threonine metabolism |
| cg17427781 | 1.12E-02 | 1.63E-03 | 5.44E-11 | 9.96E-06 | 1 | *DDX20* | TSS200 | Regulates RNA transport |
| cg15571277 | 6.29E-03 | 9.18E-04 | 7.15E-11 | 1.05E-05 | 20 | *FLJ16779* | TSS200 |  |
| cg00129563 | 1.61E-02 | 2.39E-03 | 1.23E-10 | 1.50E-05 | 5 | *PRDM6* | Body | Negative regulation of transcription, DNA-templated,negative regulation of smooth muscle cell differentiation |
| cg11179286 | 2.62E-02 | 4.01E-03 | 4.74E-10 | 4.95E-05 | 13 | *UPF3A* | Body | Nuclear-transcribed mRNA catabolic process, nonsense-mediated decay,nucleocytoplasmic transport,positive regulation of translation,mRNA transport |
| cg27266176 | 3.19E-02 | 5.01E-03 | 1.13E-09 | 9.21E-05 | 14 | *PLEKHG3* | TSS1500 | Regulation of Rho protein signal transduction,positive regulation of GTPase activity |
| cg22705016 | 3.66E-02 | 5.75E-03 | 1.06E-09 | 9.21E-05 | 2 |  | IGR |  |
| cg18489012 | 6.73E-03 | 1.07E-03 | 1.60E-09 | 1.17E-04 | 6 | *SFRS3* | TSS200 |  |
| cg12646385 | 1.24E-02 | 2.00E-03 | 2.56E-09 | 1.70E-04 | 5 |  | IGR |  |
| cg05796321 | 1.58E-02 | 2.54E-03 | 2.80E-09 | 1.71E-04 | 1 | *GFI1* | 5'UTR | Negative regulation of vitamin D biosynthetic process,negative regulation of neuron projection development,negative regulation of NF-kappaB transcription factor activity,regulation of toll-like receptor signaling pathway,negative regulation of transcription, DNA-templated,positive regulation of interleukin-6-mediated signaling pathway,cellular response to lipopolysaccharide |
| cg22788606 | 1.05E-02 | 1.70E-03 | 3.46E-09 | 1.95E-04 | 1 | *CD247* | Body | Natural killer cell mediated cytotoxicity,T cell receptor signaling pathway,Chagas disease |
| cg14935078 | 1.53E-02 | 2.63E-03 | 1.85E-08 | 9.66E-04 | 19 | *ABHD8* | TSS200 | Extracellular exosome |
| cg11728497 | 1.51E-02 | 2.63E-03 | 2.97E-08 | 1.38E-03 | 12 | *CDKN1B* | 3'UTR | Epstein-Barr virus infection,Pathways in cancer |
| cg26156007 | 1.27E-02 | 2.21E-03 | 3.02E-08 | 1.38E-03 | 7 |  | IGR |  |
| cg12225226 | 1.53E-02 | 2.68E-03 | 3.45E-08 | 1.40E-03 | 13 |  | IGR |  |
| cg24774271 | -3.07E-02 | 5.36E-03 | 3.36E-08 | 1.40E-03 | 12 |  | IGR |  |
| cg04783204 | 6.54E-03 | 1.16E-03 | 4.87E-08 | 1.78E-03 | 6 | *SLC29A1* | 5'UTR | Nucleobase-containing compound metabolic process,cellular response to glucose stimulus,cellular response to hypoxia,nucleoside transmembrane transport |
| cg18603396 | 2.79E-02 | 5.02E-03 | 7.59E-08 | 2.65E-03 | 11 | *CADM1* | TSS200 | Liver development,T cell mediated cytotoxicity,immune system process,apoptotic process,homophilic cell adhesion via plasma membrane adhesion molecules,brain development,cell differentiation,susceptibility to natural killer cell mediated cytotoxicity |
| cg25173039 | 1.23E-02 | 2.22E-03 | 8.20E-08 | 2.73E-03 | 14 | *CIPC* | TSS200 | Negative regulation of circadian rhythm,negative regulation of transcription, DNA-templated,rhythmic process |
| cg27143824 | 1.24E-02 | 2.24E-03 | 8.62E-08 | 2.74E-03 | 4 | *C4orf41* | 5'UTR |  |
| cg14898140 | 7.46E-03 | 1.37E-03 | 1.30E-07 | 3.96E-03 | 17 | *KPNA2* | 5'UTR | Regulation of DNA recombination,DNA metabolic process,protein import into nucleus,modulation by virus of host process,intracellular transport of virus |
| cg05568761 | 1.40E-02 | 2.57E-03 | 1.51E-07 | 4.43E-03 | 17 | *UBTF* | TSS1500 | Regulation of transcription from RNA polymerase I promoter,regulation of glucose mediated signaling pathway |
| cg09951570 | 5.94E-03 | 1.11E-03 | 2.20E-07 | 6.18E-03 | 14 | *C14orf147* | TSS1500 |  |
| cg08743881 | 9.40E-03 | 1.76E-03 | 2.42E-07 | 6.57E-03 | 17 | *NLK* | TSS1500 | MAPK cascade,regulation of transcription, DNA-templated,protein phosphorylation,transforming growth factor beta receptor signaling pathway,Wnt signaling pathway, calcium modulating pathway |
| cg18310007 | 4.53E-03 | 8.53E-04 | 2.64E-07 | 6.67E-03 | 17 | *EME1* | 5'UTR | Resolution of meiotic recombination intermediates,DNA repair,double-strand break repair,replication fork processing |
| cg03713379 | 4.86E-03 | 9.15E-04 | 2.58E-07 | 6.67E-03 | 5 |  | IGR |  |
| cg11813617 | 7.02E-03 | 1.34E-03 | 3.60E-07 | 8.79E-03 | 7 | *KRIT1* | TSS1500 | Negative regulation of endothelial cell proliferation,small GTPase mediated signal transduction,negative regulation of endothelial cell migration,negative regulation of angiogenesis,negative regulation of endothelial cell apoptotic process |
| cg06755612 | 9.63E-03 | 1.84E-03 | 3.95E-07 | 9.32E-03 | 14 | *FKBP3* | TSS200 | Protein peptidyl-prolyl isomerization,chaperone-mediated protein folding |
| cg01680773 | 6.03E-03 | 1.16E-03 | 4.23E-07 | 9.67E-03 | 14 | *NPC2* | TSS200 | Cholesterol metabolic process,response to virus,phospholipid transport,regulation of isoprenoid metabolic process,glycolipid transport |
| cg08679808 | 1.27E-02 | 2.45E-03 | 4.76E-07 | 1.06E-02 | 17 | *LOC92659* | Body |  |
| cg19643841 | 8.24E-03 | 1.59E-03 | 5.18E-07 | 1.11E-02 | 6 | *HMGN3* | 5'UTR | Positive regulation of transcription from RNA polymerase II promoter,positive regulation of sequence-specific DNA binding transcription factor activity,regulation of insulin secretion involved in cellular response to glucose stimulus |
| cg15416329 | 1.21E-02 | 2.35E-03 | 5.56E-07 | 1.16E-02 | 14 | *VSX2* | TSS200 | Regulation of transcription, DNA-templated,multicellular organism development,visual perception,response to stimulus |
| cg26450740 | 1.72E-02 | 3.36E-03 | 6.40E-07 | 1.30E-02 | 10 | *C10orf93* | TSS200 |  |
| cg10569335 | 1.92E-02 | 3.81E-03 | 9.53E-07 | 1.89E-02 | 5 | *RNU5E* | Body |  |
| cg10481660 | 1.57E-02 | 3.14E-03 | 1.26E-06 | 2.36E-02 | 7 | *EN2* | TSS1500 | Negative regulation of neuron apoptotic process,positive regulation of transcription from RNA polymerase II promoter,neuron development,embryonic brain development |
| cg04794832 | 4.59E-03 | 9.26E-04 | 1.46E-06 | 2.68E-02 | 17 | *RHBDL3* | TSS200 | Involved in protein procession |
| cg08759960 | 1.07E-02 | 2.19E-03 | 2.08E-06 | 3.71E-02 | 6 | *LY6G5C* | TSS1500 | Integral component of membrane |
| cg06791473 | 8.27E-03 | 1.70E-03 | 2.24E-06 | 3.91E-02 | 12 | *RND1* | 5'UTR | Negative regulation of cell adhesion,small GTPase mediated signal transduction,neuron remodeling |
| cg23097564 | 6.21E-03 | 1.28E-03 | 2.53E-06 | 3.96E-02 | 2 | *MKI67IP* | Body |  |
| cg12232388 | 1.10E-02 | 2.29E-03 | 2.60E-06 | 3.96E-02 | 12 |  | IGR |  |
| cg04351668 | 1.73E-02 | 3.57E-03 | 2.45E-06 | 3.96E-02 | 12 | *MARCH9* | Body | Protein ubiquitination |
| cg19021318 | 1.40E-02 | 2.92E-03 | 3.25E-06 | 4.62E-02 | 6 | *TUBB* | Body | Movement of cell or subcellular component,microtubule-based process,cellular process,cytoskeleton-dependent intracellular transport,natural killer cell mediated cytotoxicity,spindle assembly,cell division |
| cg17498773 | 8.81E-03 | 1.85E-03 | 3.56E-06 | 4.83E-02 | 11 | *RAB39* | Body |  |

DMPs, differential methylated probes; FDR, false discovery rate; SE, standard error; SSBC, Shanghai sleep birth cohort.

^a^ Adjusted for maternal age, education level, pre-pregnancy BMI, gestational weight gain, offspring sex, batch effect, and cell type.

Gene function^*^:https://david.ncifcrf.gov/.

**Table S3 The sex-specific association between each maternal sleep parameter and the offspring adiposity indicators at 2 years old in total sample**

|  | BMI (N = 2167) | | | | |  | SF (N = 1493) | | | | |
| --- | --- | --- | --- | --- | --- | --- | --- | --- | --- | --- | --- |
|  | Male (N = 1116) | | Female (N = 1051) | |  |  | Male (N = 780) | | Female (N = 713) | |  |
|  | Coef. (95% CI) | *p* | Coef. (95% CI) | *p* | *P*^a^ |  | Coef. (95% CI) | *p* | Coef. (95% CI) | *p* | *P*^b^ |
| **Model 1** |  |  |  |  |  |  |  |  |  |  |  |
| Nighttime sleep duration | 0.05 (-0.02, 0.13) | 0.144 | 0.05 (-0.02, 0.12) | 0.180 | 0.951 |  | -0.19 (-0.43, 0.05) | 0.113 | 0.03 (-0.25, 0.31) | 0.853 | 0.240 |
| Sleep quality |  |  |  |  |  |  |  |  |  |  |  |
| Good | Ref. |  | Ref. |  |  |  | Ref. |  | Ref. |  |  |
| Poor | -0.06 (-0.26, 0.14) | 0.564 | -0.09 (-0.28, 0.09) | 0.333 | 0.816 |  | 0.29 (-0.40, 0.98) | 0.410 | -0.06 (-0.81, 0.69) | 0.876 | 0.501 |
| Sleep midpoint | -0.05 (-0.15, 0.05) | 0.335 | 0.01 (-0.10, 0.11) | 0.901 | 0.455 |  | **0.47 (0.14, 0.79)** | **0.005** | **0.75 (0.34, 1.15)** | **<0.001** | 0.288 |
| **Model 2** |  |  |  |  |  |  |  |  |  |  |  |
| Nighttime sleep duration | 0.06 (-0.01, 0.13) | 0.099 | 0.03 (-0.05, 0.10) | 0.480 | 0.802 |  | -0.08 (-0.32, 0.16) | 0.526 | -0.00 (-0.29, 0.28) | 0.976 | 0.330 |
| Sleep quality |  |  |  |  |  |  |  |  |  |  |  |
| Good | Ref. |  | Ref. |  |  |  | Ref. |  | Ref. |  |  |
| Poor | -0.01 (-0.21, 0.19) | 0.911 | -0.08 (-0.27, 0.10) | 0.376 | 0.621 |  | 0.32 (-0.36, 0.99) | 0.360 | -0.15 (-0.88, 0.59) | 0.691 | 0.342 |
| Sleep midpoint | -0.04 (-0.14, 0.05) | 0.397 | 0.00 (-0.10, 0.10) | 0.985 | 0.630 |  | **0.49 (0.17, 0.81)** | **0.002** | **0.78 (0.39, 1.18)** | **<0.001** | 0.285 |

BMI, body mass index; SF, subcutaneous fat.

Ref.: women with good sleep quality were selected as reference group.

^a^ The statistical test of the interaction effect of maternal sleep and offspring sex on BMI at 2 years old.

^b^ The statistical test of the interaction effect of maternal sleep and offspring sex on SF at 2 years old.

Model 1: unadjusted.

Model 2: adjusted for maternal age, education level, pre-pregnancy BMI, and gestational weight gain.

**Table S4 The sensitivity analysis of the association between each maternal sleep parameter and the offspring adiposity indicators at 2 years old in total sample^a^**

|  | BMI (N = 2162) | |  | SF (N = 1490) | |
| --- | --- | --- | --- | --- | --- |
|  | Coef. (95% CI) | *p* |  | Coef. (95% CI) | *p* |
| **Model 1** |  |  |  |  |  |
| Nighttime sleep duration | 0.05 (-0.00, 0.10) | 0.061 |  | -0.06 (-0.25, 0.13) | 0.517 |
| Sleep quality |  |  |  |  |  |
| Good | Ref. |  |  | Ref. |  |
| Poor | -0.10 (-0.24, 0.03) | 0.135 |  | 0.07 (-0.44, 0.58) | 0.785 |
| Sleep midpoint | -0.02 (-0.09, 0.06) | 0.671 |  | **0.61 (0.36, 0.87)** | **<0.001** |
| **Model 2** |  |  |  |  |  |
| Nighttime sleep duration | 0.04 (-0.01, 0.10) | 0.116 |  | 0.01 (-0.18, 0.20) | 0.902 |
| Sleep quality |  |  |  |  |  |
| Good | Ref. |  |  | Ref. |  |
| Poor | -0.06 (-0.19, 0.08) | 0.410 |  | 0.06 (-0.44, 0.56) | 0.811 |
| Sleep midpoint | -0.02 (-0.10, 0.05) | 0.491 |  | **0.63 (0.38, 0.88)** | **<0.001** |

**^a^** The model was conducted among participants whose NSD with -4SD~+4SD.

BMI, body mass index; SF, subcutaneous fat.

Ref.: women with good sleep quality were selected as reference group.

Model 1: unadjusted.

Model 2: adjusted for maternal age, education level, pre-pregnancy BMI, gestational weight gain, and offspring’s sex.

**Table S5 The association between the maternal sleep parameters during late pregnancy and the offspring adiposity indicators at 2 years old in the total sample after multiple imputation (N = 2211)**

|  | BMI | |  | SF | |
| --- | --- | --- | --- | --- | --- |
|  | Coef. (95% CI) | *p* |  | Coef. (95% CI) | *p* |
| **Model 1** |  |  |  |  |  |
| Nighttime sleep duration | **0.05 (0.00, 0.11)** | **0.038** |  | -0.07 (-0.26, 0.12) | 0.466 |
| Sleep quality |  |  |  |  |  |
| Good | Ref. |  |  | Ref. |  |
| Poor | -0.10 (-0.24, 0.04) | 0.155 |  | 0.11 (-0.41, 0.63) | 0.684 |
| Sleep midpoint | -0.01 (-0.09, 0.06) | 0.703 |  | **0.61 (0.34, 0.87)** | **<0.001** |
| **Model 2** |  |  |  |  |  |
| Nighttime sleep duration | 0.05 (-0.01, 0.10) | 0.078 |  | -0.02 (-0.21, 0.17) | 0.809 |
| Sleep quality |  |  |  |  |  |
| Good | Ref. |  |  | Ref. |  |
| Poor | -0.05 (-0.19, 0.08) | 0.437 |  | 0.11 (-0.40, 0.62) | 0.675 |
| Sleep midpoint | -0.03 (-0.10, 0.04) | 0.480 |  | **0.61 (0.35, 0.87)** | **<0.001** |

BMI, body mass index; SF, subcutaneous fat.

Ref.: women with good sleep quality were selected as reference group.

Model 1: unadjusted.

Model 2: adjusted for maternal age, education level, pre-pregnancy BMI, gestational weight gain, and offspring’s sex.

**Table S6 The sex-specific association between each maternal sleep parameter and the offspring adiposity indicators at 2 years old in total sample after multiple imputation (N = 2211)**

|  | BMI | | | | |  | SF | | | | |
| --- | --- | --- | --- | --- | --- | --- | --- | --- | --- | --- | --- |
|  | Male | | Female | |  |  | Male | | Female | |  |
|  | Coef. (95% CI) | *p* | Coef. (95% CI) | *p* | *P*^a^ |  | Coef. (95% CI) | *p* | Coef. (95% CI) | *p* | *P*^b^ |
| **Model 1** |  |  |  |  |  |  |  |  |  |  |  |
| Nighttime sleep duration | 0.05 (-0.02, 0.13) | 0.141 | 0.05 (-0.02, 0.12) | 0.174 | 0.956 |  | -0.16 (-0.40, 0.09) | 0.209 | 0.02 (-0.24, 0.29) | 0.857 | 0.305 |
| Sleep quality |  |  |  |  |  |  |  |  |  |  |  |
| Good | Ref. |  | Ref. |  |  |  | Ref. |  | Ref. |  |  |
| Poor | -0.06 (-0.26, 0.14) | 0.579 | -0.09 (-0.28, 0.09) | 0.328 | 0.799 |  | 0.20 (-0.47, 0.87) | 0.553 | 0.07 (-0.66, 0.81) | 0.849 | 0.786 |
| Sleep midpoint | -0.04 (-0.14, 0.05) | 0.364 | 0.01 (-0.10, 0.11) | 0.868 | 0.461 |  | **0.51 (0.18, 0.84)** | **0.002** | **0.71 (0.31, 1.10)** | **<0.001** | 0.432 |
| **Model 2** |  |  |  |  |  |  |  |  |  |  |  |
| Nighttime sleep duration | 0.06 (-0.01, 0.13) | 0.102 | 0.03 (-0.05, 0.10) | 0.473 | 0.800 |  | -0.06 (-0.31, 0.19) | 0.646 | -0.12 (-0.29, 0.25) | 0.903 | 0.434 |
| Sleep quality |  |  |  |  |  |  |  |  |  |  |  |
| Good | Ref. |  | Ref. |  |  |  | Ref. |  | Ref. |  |  |
| Poor | -0.01 (-0.21, 0.18) | 0.894 | -0.09 (-0.27, 0.10) | 0.359 | 0.619 |  | 0.23 (-0.43, 0.89) | 0.501 | -0.00 (-0.72, 0.72) | 0.994 | 0.588 |
| Sleep midpoint | -0.04 (-0.14, 0.06) | 0.415 | 0.00 (-0.10, 0.10) | 0.993 | 0.556 |  | **0.54 (0.22, 0.87)** | **0.001** | **0.72 (0.33, 1.11)** | **<0.001** | 0.441 |

BMI, body mass index; SF, subcutaneous fat.

Ref.: women with good sleep quality were selected as reference group.

^a^ The statistical test of the interaction effect of maternal sleep and offspring sex on BMI at 2 years old.

^b^ The statistical test of the interaction effect of maternal sleep and offspring sex on SF at 2 years old.

Model 1: unadjusted.

Model 2: adjusted for maternal age, education level, pre-pregnancy BMI, and gestational weight gain.

**Table S7 The sensitivity analysis of the association between each maternal sleep parameter and the offspring adiposity indicators at 2 years old in total sample^a^ after imputation (N = 2206)**

|  | BMI | |  | SF | |
| --- | --- | --- | --- | --- | --- |
|  | Coef. (95% CI) | *p* |  | Coef. (95% CI) | *p* |
| **Model 1** |  |  |  |  |  |
| Nighttime sleep duration | 0.05 (0.00, 0.10) | 0.058 |  | -0.06 (-0.25, 0.13) | 0.551 |
| Sleep quality |  |  |  |  |  |
| Good | Ref. |  |  | Ref. |  |
| Poor | -0.10 (-0.24, 0.03) | 0.137 |  | 0.09 (-0.43, 0.61) | 0.730 |
| Sleep midpoint | -0.01 (-0.09, 0.06) | 0.708 |  | **0.61 (0.34, 0.87)** | **<0.001** |
| **Model 2** |  |  |  |  |  |
| Nighttime sleep duration | 0.04 (-0.02, 0.10) | 0.117 |  | -0.01 (-0.20, 0.18) | 0.918 |
| Sleep quality |  |  |  |  |  |
| Good | Ref. |  |  | Ref. |  |
| Poor | -0.06 (-0.19, 0.08) | 0.399 |  | 0.10 (-0.42, 0.61) | 0.723 |
| Sleep midpoint | -0.02 (-0.10, 0.05) | 0.504 |  | **0.62 (0.36, 0.88)** | **<0.001** |

**^a^** The model was conducted among participants whose NSD with -4SD~+4SD.

BMI, body mass index; SF, subcutaneous fat.

Ref.: women with good sleep quality were selected as reference group.

Model 1: unadjusted.

Model 2: adjusted for maternal age, education level, pre-pregnancy BMI, gestational weight gain, and offspring’s sex.


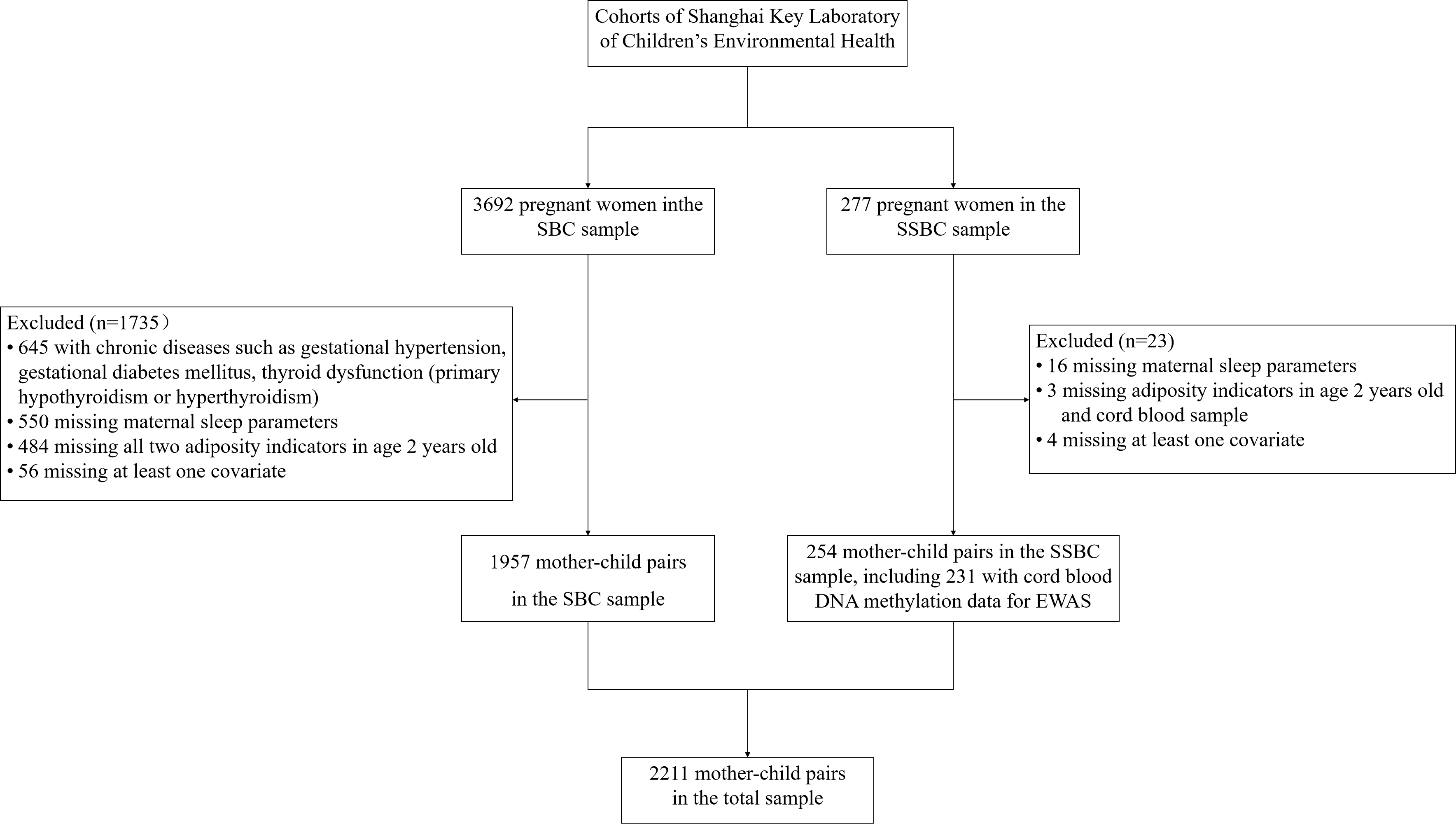


**Figure S1** **Flowchart of the participants**

EWAS, epigenome-wide association study; SBC, Shanghai birth cohort; SSBC, Shanghai sleep birth cohort.
